# Isolation and Identification of Novel Taste-Modulating *N*^2^-Guanosine 5′-Monophosphate Derivatives Generated by Maillard-Type Reactions

**DOI:** 10.1021/acs.jafc.4c03485

**Published:** 2024-06-13

**Authors:** Daniela M. Hartl, Oliver Frank, Victoria S. Hänel, Vinzenz Heigl, Corinna Dawid, Thomas F. Hofmann

**Affiliations:** † Chair of Food Chemistry and Molecular Sensory Science, Technical University of Munich, Lise-Meitner-Str. 34, 85354 Freising, Germany; ‡ Professorship for Functional Phytometabolomics, TUM School of Life Sciences, 10 Technical University of Munich, Lise-Meitner-Str. 34, D-85354 Freising, Germany

**Keywords:** guanosine 5′-monophosphate derivatives, umami, taste modulating, 2-methyl-3-furanthiol, Maillard-type model reactions

## Abstract

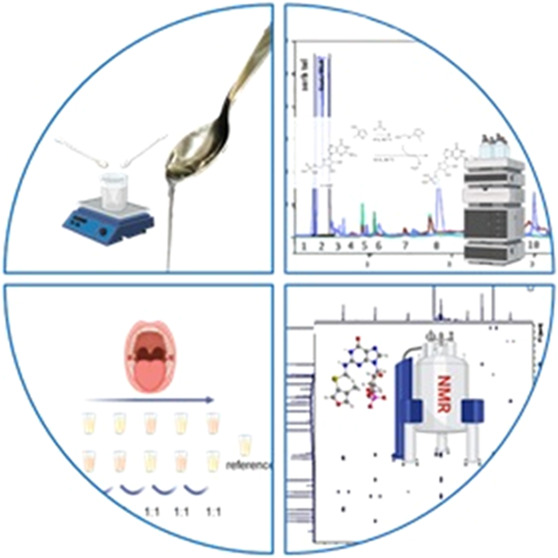

Several compounds with taste-modulating properties have been investigated, improving the taste impression without having a pronounced intrinsic taste. The best-known representatives of umami taste-modulating compounds are ribonucleotides and their derivatives. Especially the thio derivatives showed high taste-modulating potential in structure–activity relationship investigations. Therefore, this study focuses on the formation of guanosine 5′-monophosphate derivatives consisting of Maillard-type generated compounds like the aroma-active thiols (2-methyl-3-furanthiol, 3-mercapto-2-pentanone, 2-furfurylthiol) and formaldehyde to gain insights into the potential of combinations of taste and aroma-active compounds. One literature-known (*N*^2^-(furfurylthiomethyl)-guanosine 5′-monophosphate) and three new derivatives (*N*^2^-(2-methyl-1-furylthiomethyl)-guanosine 5′-monophosphate, *N*^2^-((5-hydroxymethyl)-2-methyl-1-furylthiomethyl)-guanosine 5′-monophosphate, *N*^2^-((2-pentanon-1-yl)thiomethyl)-guanosine 5′-monophosphate) were successfully produced using green natural deep eutectic solvents and isolated, and their structures were completely elucidated. Besides the intrinsic taste properties, the kokumi and umami taste-modulating effects of the four derivatives were evaluated via psychophysical investigations, ranging from 19 to 22 μmol/L.

## Introduction

New taste-modulating compounds can ensure healthy, environmentally friendly food without limitations in taste. High sodium chloride (NaCl) consumption, for example, is associated with health risks like cardiovascular diseases, hypertension, strokes, and stomach cancer.^1−3^ A decrease in the current estimated daily NaCl intake of 9–12 g in industrialized countries to the recommended limit of 5 g per day (World Health Organization (WHO)) could decrease the risk of these diseases.^4^ Besides NaCl, the European Food Safety Association (EFSA) specified a group acceptable daily intake (ADI) of 30 mg/kg for l-glutamate and l-glutamic acid and a no-observed adverse effect level (NOAEL) for monosodium l-glutamate (MSG) of 3200 mg/kg referred to the individual body weight. Nearly all population groups exceed this ADI.^5,6^ In general, a relationship between NaCl and MSG concentrations and palatability was demonstrated in 1984 by Yamaguchi and Takahashi.^7^ MSG is associated with the taste quality of umami, first described in 1908 by Ikeda.^8^ As reported in 1866 by Fischer,^9^ the taste activity of MSG and other salts of l-glutamic acid showed a pH dependency with a weak sour and insipid taste.^8−11^ After Ikeda’s investigation on MSG,^8^ more umami-tasting compounds were discovered, like the free amino acids l-aspartic acid, l-glutamine, and l-asparagine,^12^ amino acid derivatives like glutamate glycoconjugates,^13^ succinic acid,^14^ or different compounds, e.g., from morel mushrooms the glucopyranoside of (*S*)-malic acid, (*S*)-morelid.^15^ A special feature of umami is the synergistic enhancing effect of ribonucleotides like the disodium salts of inosine 5′-monophosphate (IMP) and guanosine 5′-monophosphate (GMP) in combination with MSG.^16−18^ Yamaguchi^16^ showed in 1967 that this synergistic enhancing effect has the highest impact in a proportion range of 30–70% of MSG to IMP.^16^ On the molecular receptor level, umami taste stimuli bind predominantly to a heterodimer of type 1 taste receptors (T1R1 and T1R3) of G protein-coupled receptors (GPCRs).^19^ In addition to T1R1/T1R3, other receptors for umami taste perception, such as the metabotropic glutamate receptors (mGluRs), are known.^11,17,18,20−23^ The T1R1/T1R3 receptor has two large N-terminal extracellular domains with a bilobed structure called the Venus flytrap domain (VFT).^20−22^ The binding of l-glutamate to the VFT transforms the receptor from an open to a closed conformation.^18,21^ Li et al. showed that IMP and GMP alone did not activate the T1R1/T1R3 receptor, but the adjacent binding of IMP to l-glutamate stabilizes the closed conformation of the VFT of T1R1.^18,22^ Zhang et al. described this stabilizing effect as a positive allosteric modulation of the umami taste.^18^ Therefore, the 5′-ribonucleotides and their derivatives are positive, allosteric taste-modulating compounds for the umami taste. In general, taste-modulating compounds, in combination with other substances, significantly enhance or decrease the taste impression while they have less or no intrinsic taste.^24^ Depending on their receptor binding site, these taste modulators can be divided into positive and negative and orthosteric and allosteric modulators.^17,20^ In addition to IMP and GMP, numerous derivatives have been investigated in structure–activity studies for a positive modulatory effect using comparative psychophysical experiments.^25−30^ The resulting so-called β-value referred to the enhancing effect of IMP (1.0) and was established by Yamaguchi et al.^30^ Imai et al. demonstrated that the insertion of sulfur atoms at the appropriate position two leads to higher β-values and, therefore, to higher synergistic effects.^29^ The synthesis and psychophysical evaluation of 33 different compounds of two substituted IMP derivatives showed comparable 2-O- and 2-N-substituted compounds exhibited lower β-values than their 2-S-substituted analogues. Interestingly, derivatives with sulfonic acid and dithiol groups had nearly no impact on the synergistic umami taste. The synthesized 2-furfurylthioinosine 5′-monophosphate showed the highest β-value of 17.3.^29^ Cairoli et al. and Morelli et al. studied the synergistic effect of different *N*^2^-alkyl and *N*^2^-acyl GMP derivatives.^26,28^ The sensory activity depends on the chain length and the substituent. A C_4_*N*^2^-alkyl GMP derivate resulted in a β-value of 4.1, and replacing the third methylene group in the alkyl chain by a sulfur atom increased the β-value to 4.6 and an additional sulfur atom between the C_3_ and the C_4_ increased it to 5.7.^26^ A sulfoxide group instead of the third methylene group within the alkyl chain resulted in a decrease to 2.9 (Figure S1, Supporting Information).^28^ Based on these observations, Suess et al. synthesized 13 different *N*^2^-alkylthiomethyl- and *N*^2^-arylthiomethyl-GMP derivatives by using the Maillard reaction product formaldehyde as an electrophilic linker between the GMP moiety and the thiol.^27^ Therefore, the generation of the *N*^2^-(propylthiomethyl)guanosine 5′-monophosphate was also possible with the educts GMP, glucose, glycine, and the aroma compound 1-propanethiol, which is naturally present in onions. Generally, formaldehyde occurs in foods and the human body as a Maillard reaction product due to the Strecker degradation of the amino acid glycine.^27,31^ Suess et al. used the naturally occurring kokumi taste-modulating glutathione or the aroma compound 2-furfurylthiol (FFT) for other model reactions.^27^ Brehm et al. demonstrated that aroma-active thiols like FFT, 2-methyl-3-furanthiol (MFT), or 3-mercapto-2-pentanone (MP) coupled to thiamine-derived pyrimidine moieties increased the kokumi taste impression,^32^ which is described to enhance mouthfulness, continuity, richness, complexity, and thickness of umami-tasting solutions,^33−35^ in savory foods.

Therefore, this study aimed to generate, isolate, and investigate GMP derivatives with the aroma-active thiols MFT and MP that occur naturally, e.g., in yeast extract^36^ or heated meat,^37−39^ and to investigate their taste-enhancing potential by determining their β-values and their intrinsic taste and taste-modulating thresholds. The potentially new taste-modulating compounds should be produced by model reactions which may occur during food processing or preparation in a natural deep eutectic solvent (NADES) consisting of sucrose and d-sorbitol, which has recently been tested for its suitability for the production of such *N*^2^-substituted GMP derivatives.^40,41^ In general, NADES systems show, besides benefits like low- or nontoxicity, biodegradability, and low water content, high solubility capacity for the formation of highly concentrated reaction systems promising high yields of target compounds, which was recently tested for the suitability of Maillard-type reactions of nucleotide derivates.^41−43^

## Materials and Methods

### Chemicals

The listed chemicals were obtained from commercial sources: sucrose (≥99.5%), d-sorbitol (≥98.0%), d-sorbitol (food-grade), guanosine 5′-monophosphate (GMP) disodium salt hydrate (≥99.0%), inosine 5′-monophosphate (IMP) from *Saccharomyces cerevisiae* (≥98.0%), formic acid (98.0–100.0%), maltodextrin (16.5–19.5 dextrose equivalent), NaCl (99.0–100.0%), sodium l-glutamate monohydrate, lactic acid, reduced glutathione, 2-methyl-3-furanthiol (MFT; ≥95.0%), and formaldehyde (36.5–38.0% in water) and the deuterated chemicals deuterium oxide (D_2_O), methanol-*d*_4_, and 3-(Trimethylsilyl)propionic-2,2,3,3-*d*_4_ acid sodium salt (TMSP) from Merck KGaA (Darmstadt, Germany); tyrosine (≥99.0%) from Fluka (Buchs, St. Gallen, Switzerland); 2-furfurylthiol (FFT; ≥98.0%) from Thermo Fisher Scientific GmbH (Dreieich, Germany); caffeine (99.0%) and sucrose (food-grade) (99.0%) from Alfa Aesar (Kandel, Germany), GMP (≥98.0%) and 2-mercapto-3-pentanone (MP; (*R*)-, (*S*)-mixture; 98.0%) from Abcr GmbH (Karlsruhe, Germany); formic acid (98–100%), formic acid (mass spectrometry (MS) grade), and ethyl acetate (≥99.5%) from VWR (Darmstadt, Germany). For sensory experiments, Gistex XII yeast extract from DSM (Heerlen, Nederlands) and natural mineral water from Evian (Évian-les-Bains, France) were used. Water for the mobile phase of UHPLC or HPLC separations was deionized and purified by using the Milli-Q reference A+ system from Merck Millipore (Darmstadt, Germany), and acetonitrile HPLC-grade and UHPLC-MS-grade was purchased from Thermo Fisher Scientific GmbH (Dreieich, Germany). The NADES system as a reaction medium was prepared as recently published in the literature.^41,42^

### Model Reactions and Isolation of the Reaction Products by HPLC

#### Generation of Taste-Modulating *N*^2^-(Alkylthiomethyl)- and *N*^2^-(Arylthiomethyl)-GMP Derivatives

The generation of different GMP derivatives was implemented in a sucrose/d-sorbitol/water (1:1:8) NADES system according to the generation of *N*^2^-(furfurylthiomethyl) guanosine 5′-monophosphate (**1**)^41^ with some modifications based on the literature (Figure S2, Supporting Information).^27,43^ Each active aroma thiol (MFT, MP, FFT) (0.30 mmol) was mixed with the NADES system (1.0 g) spiked with formaldehyde solution (0.75 mmol, 23 μL of a 37% solution in water) and heated under stirring for four h at 40 °C. The target compounds were generated by adding GMP (0.30 mmol). After heating for 16 h at 40 °C, the reaction was stopped by the addition of water (10 mL). A membrane-filtered (0.45 μm) aliquot was then separated by reversed-phase HPLC combined with an ultraviolet/visible light detector (RP-HPLC-UV/vis) (two pumps P 6.1L, detector MWD 2.1L, fraction collector: LABOCOL Vario-4000, software PurityChrom Version 5.09.036; Knauer Wissenschaftliche Geräte GmbH, Berlin, Germany). A wavelength of 260 nm and an injection volume of 1–2 mL were used for preparative separations. Chromatography was performed for all reaction mixtures in the first separation step by using a Luna pentafluorophenyl (PFP) column (250 mm × 21.2 mm, 100 Å, 5.0 μm) with a corresponding guard column (Phenomenex, Aschaffenburg, Germany) as the stationary phase, and a mixture of acetonitrile and water each with 0.1% formic acid at a flow rate of 20 mL/min was used as the mobile phase. Chromatographic separation of the reaction mixture of GMP, formaldehyde, and MFT was achieved with the following gradient: starting at 0% B for 3.0 min; increasing within 17.0 min to 35% B; increasing within 2.0 min to 100% B and maintaining 100% B for 3.0 min. After separation and lyophilization, out of the eight fractions obtained, fractions five (retention time (RT): 12.0 min) and six (RT: 14.0 min) were further purified (Figure S3, Supporting Information).

For purification of fraction six, the same Luna PFP column was used with the following gradient and a flow rate of 20 mL/min of a solvent mixture of acetonitrile and water: starting at 0% B for 5.0 min, increasing to 30% B within 12.0 min; increasing to 80% B within 3.0 min and holding 100% B for further 2.0 min. The structure of the compound eluting at an RT of 10.0 min was identified as *N*^2^*-(*2-Methyl-1-furylthiomethyl)-guanosine 5′-monophosphate (**2**) using Ultraperformance Liquid Chromatography Time-of-Flight Mass Spectrometer (UHPLC-ToF-MS) and nuclear magnetic resonance spectroscopy (NMR) measurements. Purification of fraction five was performed with a Luna PFP column (250 mm × 10.0 mm, 100 Å, 5 μm) with a corresponding guard column, an injection volume of 300 μL, and a flow rate of 4.8 mL/min. The following gradient, starting at 0% B for 3.0 min, increasing B to 35% within 8.0 min, increasing within 4.0 min to 80% B, and maintaining 80% B for 3.0 min, was used for separation (Figure S4, Supporting Information). After lyophilization, the isolated compound at an RT of 14.0 min was identified as *N*^2^*-*((5-Hydroxymethyl)-2-methyl-1-furylthiomethyl)-guanosine 5′-monophosphate (**3**) by LC-ToF-MS and NMR measurements. For the separation of the reaction products of GMP, formaldehyde, and MP, the following gradient was used in the first separation step: starting with 0% B for 10.0 min, increasing within 13.0 min to 30% B, increasing within 2.0 min to 100% B; holding 100% B for 3.0 min (Figure S5, Supporting Information). The solvent of each fraction was removed by evaporation and lyophilization (Martin Christ Gefriertrocknungsanlagen GmbH, Osterode, Germany). From fraction six out of the seven obtained fractions, the product was isolated by using a semipreparative NUCLEODUR C18-Pyramid column (250.0 mm × 10.0 mm, 100 Å, 5 μm, Macherey & Nagel, Düren, Germany) with a corresponding guard column at a flow rate of 4.8 mL/min. The gradient started with 0% B for 7.0 min, increased within 8.0 min to 35% B, and was maintained for 5.0 min (Figure S6, Supporting Information). After lyophilization, fractions four out of the five observed fractions could be identified as *N*^2^*-*((2-Pentanon-1-yl)thiomethyl)-guanosine 5′-monophosphate (**4**) by UHPLC-ToF-MS and NMR measurements.

The parameters for the separation and characterization of *N*^2^-(furfurylthiomethyl)-guanosine 5′-monophosphate (**1**) can be taken from the publication by Suess et al.^41^

#### *N*^2^*-(*2-Methyl-1-furylthiomethyl)-guanosine 5′-Monophosphate (**2**)

UV/vis (water/acetonitrile, 70:30, v/v; 0.1% formic acid): λ_max_ = 264 nm. UHPLC-TOF-MS (ESI^–^) *m*/*z* 488.0637 ([M – H]^−^, measured); *m*/*z* 488.0647 ([M – H]^−^, calcd for C_16_H_19_N_5_O_9_PS^–^). UHPLC-TOF-MS (ESI^+^) *m*/*z* 490.0789 ([M + H]^+^, measured); *m*/*z* 490.0792 ([M + H]^+^, calcd for C_16_H_21_N_5_O_9_PS^+^). ^1^H NMR (500.13 MHz, methanol-*d*_4_, 298 K, COSY) δ (ppm): 2.28 [s, 3H, H–C(6″)], 4.11–4.21 [m, 2H, H–C(5′)], 4.21–4.25 [m, 1H, H–C(4′)], 4.38 [t, *J* = 4.3 Hz, 1H, H–C(3′)], 4.59 [d, *J* = 13.4 Hz, 1H, H–C(1‴_α_)], 4.62 [t, *J* = 5.2 Hz, 1H, H–C(2′)], 4.72 [d, *J* = 13.4 Hz, 1H, H–C(1‴_β_)], 5.93 [d, *J* = 5.2 Hz, 1H, H–C(1′)], 6.43 [d, *J* = 1.9 Hz, 1H, H–C(5″)], 7.34 [d, *J* = 1.9 Hz, 1H, H–C(4″)], 8.25 [s, 1H, H–C(8)]. ^13^C NMR (125 MHz, methanol-*d*_4_, 298 K, HMBC, HSQC) δ (ppm) 11.7 [C(6″)], 47.5 [C(1‴)], 64.7 [d, *J*_C,P_ = 5.3 Hz, C(5′)], 71.9 [C(3′)], 75.9 [C(2′)], 85.2 [d, *J*_C,P_ = 8.5 Hz, C(4′)], 89.5 [C(1′)], 110.0 [C(1″)], 116.5 [C(5″)], 116.7 [C(5)], 138.1 [C(8)], 142.3 [C(4″)], 152.1 [C(4)], 153.4 [C(2)], 157.2 [C(2″)], 158.5 [C(6)].

#### *N*^2^*-*((5-Hydroxymethyl)-2-methyl-1-furylthiomethyl)-guanosine 5′-Monophosphate (**3**)

UV/vis (water/acetonitrile, with 0.1% formic acid added, 70:30, v/v) λ_max_ = 264 nm. UHPLC-TOF-MS (ESI^–^) *m*/*z* 518.0747 ([M – H]^−^, measured); *m*/*z* 518.0752 ([M – H]^−^, calcd for C_17_H_21_N_5_O_10_PS^–^). UHPLC-TOF-MS (ESI^+^) *m*/*z* 520.0898 ([M + H]^+^, measured); *m*/*z* 520.0898 ([M + H]^+^, calcd for C_17_H_23_N_5_O_10_PS^+^). ^1^H NMR (500.13 MHz, methanol-*d*_4_, 298 K, COSY) δ (ppm) 2.26 [s, 3H, H–C(6″)], 4.12–4.21 [m, 1H, H–C(5′_α_)], 4.21–4.30 [m, 2H, H–C(4′), H–C(5′_β_)], 4.37 [t, *J* = 4.7 Hz, 1H, H–C(3′)], 4.53 [s, 2H, H–C(7″)], 4.58 [t, *J* = 4.9 Hz, 1H, H–C(2′)], 4.62 [d, *J* = 13.4 Hz, 1H, H–C(1‴_α_)], 4.71 [d, *J* = 13.4 Hz, 1H, H–C(1‴_β_)], 5.92 [d, *J* = 4.6 Hz, 1H, H–C(1′)], 7.40 [s, 1H, H–C(4″)], 8.34 [s, 1H, H–C(8)]. ^13^C NMR (125 MHz, methanol-*d*_4_, 298 K, HMBC, HSQC) δ 12.0 [C(6″)], 48.0 [C(1‴)], 55.5 [C(7″)], 66.3 [d, *J*_C,P_ = 5.0 Hz, C(5′)], 71.5 [C(3′)], 75.9 [C(2′)], 84.9 [d, *J*_C,P_ = 8.5 Hz, C(4′)], 89.9 [C(1′)], 110.3 [C(1″)], 115.8 [C(5)], 129.4 [C(5″)], 137.8 [C(8)], 140.5 [C(4″)], 151.8 [C(4)], 153.7 [C(2)], 158.0 [C(2″)], 158.9 [C(6)].

#### (*R*)-, (*S*)-*N*^2^*-*((2-Pentanon-1-yl)thiomethyl)-guanosine 5′-Monophosphate (**4**)

UV/vis (water/acetonitrile, with 0.1% formic acid added, 70:30, v/v) λ_max_ = 264 nm. UHPLC-TOF-MS (ESI^–^) *m*/*z* 492.0964 ([M – H]^−^, measured); *m*/*z* 492.0960 ([M – H]^−^, calcd for C_16_H_23_N_5_O_9_PS^–^). UHPLC-TOF-MS (ESI^+^) *m*/*z* 494.1115 ([M + H]^+^, measured); *m*/*z* 494.1105 ([M + H]^+^, calcd for C_16_H_25_N_5_O_9_PS^+^). **4A:**^1^H NMR (500.13 MHz, methanol-*d*_4_, 298 K, COSY) δ (ppm) 0.98 [t, *J* = 7.3 Hz, 3H, H–C(5″)], 1.66–1.76 [m, 1H, H–C(4″_α_)], 1.84–1.96 [m, 1H, H–C(4″_β_)], 2.28 [s, 3H, H–C(3″)], 3.55 [t, *J* = 7.5 Hz, 1H, H–C(1″)], 4.13–4.26 [m, 3H, H–C(4′), H–C(5′)], 4.37 [t, *J* = 4.6 Hz, 1H, H–C(3′)], 4.57 [d, *J* = 14.2 Hz, 1H, H–C(1‴_α_)], 4.61–4.66 [m, 3H, H–C(2′), H–C(1‴_β_)], 6.00 [d, *J* = 4.8 Hz, 1H, H–C(1′)], 8.32 [s, 1H, H–C(8)]. **4B**: ^1^H NMR (500.13 MHz, methanol-*d*_4_, 298 K, COSY) δ (ppm) 0.98 [t, *J* = 7.4 Hz, 3H, H–C(5″)], 1.66–1.76 [m, 1H, H–C(4″_α_)], 1.84–1.96 [m, 1H, H–C(4″_β_)], 2.28 [s, 3H, H–C(3″)], 3.55 [t, *J* = 7.5 Hz, 1H, H–C(1″)], 4.13–4.26 [m, 3H, H–C(4′), H–C(5′)], 4.37 [t, *J* = 4.6 Hz, 1H, H–C(3′)], 4.58 [d, *J* = 14.2 Hz, 1H, H–C(1‴_α_)], 4.61–4.67 [m, 3H, H–C(2′), H–C(1‴_β_)], 6.00 [d, *J* = 5.1 Hz, 1H, H–C(1′)], 8.32 [s, 1H, H–C(8)]. **4A:**^13^C NMR (125 MHz, methanol-*d*_4_, 298 K, HMBC, HSQC) δ (ppm) 10.7 [C(5″)], 23.5 [C(4″)], 25.4 [C(3″)], 42.3 [C(1‴)], 55.1 [C(1″)], 65.0 [d, *J*_C,P_ = 5.2 Hz, C(5′)], 70.3 [d, *J*_C,P_ = 3.5 Hz, C(3′)], 74.5 [d, *J*_C,P_ = 12.7 Hz, C(2′)], 83.7 [d, *J*_C,P_ = 8.7 Hz, C(4′)], 88.4 [d, *J*_C,P_ = 7.6 Hz, H–C(1′)], 114.9 [C(5)], 136.5 [C(8)], 150.5 [C(4)], 152.2 [C(2)], 156.8 [C(6)], 207.1 [C(2″)]. **4B**: ^13^C NMR (125 MHz, methanol-*d*_4_, 298 K, HMBC, HSQC) δ (ppm) 10.7 [C(5″)], 23.5 [C(4″)], 25.4 [C(3″)], 42.4 [C(1‴)], 55.2 [C(1″)], 65.1 [d, *J*_C,P_ = 5.2 Hz, C(5′)], 70.3 [d, *J*_C,P_ = 3.5 Hz, C(3′)], 74.5 [d, *J*_C,P_ = 12.7 Hz, C(2′)], 83.7 [d, *J*_C,P_ = 8.7 Hz, C(4′)], 88.4 [d, *J*_C,P_ = 7.6 Hz, H–C(1′)], 114.9 [C(5)], 136.5 [C(8)], 150.5 [C(4)], 152.2 [C(2)], 156.8 [C(6)], 207.1 [C(2″)].

The NMR spectra, especially the ^13^C NMR spectra, showed a double signal set, most likely corresponding to a pair of diastereomers (ratio 1:1) formed during the model reaction with the chiral MP. It was impossible to separate these diastereomers; therefore, this mixture of two compounds is referred to as compound **4** in the manuscript.

#### Extraction of Hemithioacetals as Intermediates

To verify the reaction pathway’s first step for forming **1**–**4**, the intermediates of all reaction mixtures were studied by completely dissolving each formation approach in water (10 mL) after the first reaction step. All intermediates were extracted by liquid–liquid extraction, each with ethyl acetate (3 × 10 mL). The organic phases were combined and evaporated. The residue was resolved in 400 μL (DMSO-*d*_6_) and measured by one- and two-dimensional NMR experiments. The hemithioacetals 2-furfurylthiomethanol (**1a**), 2-methyl-3-furanthiomethanol (**2a**), and 3-hydroxymethylthio-2-pentanone (**4a**) could be identified and their structures completely elucidated (Supporting Information).

### UHPLC-TOF-MS

#### Determination of Exact Mass and Mass Fragmentation by Ultraperformance Liquid Chromatography Time-of-Flight Mass Spectrometer (UHPLC-TOF-MS)

Exact mass-to-charge ratios (*m*/*z*) of the isolated compounds **1**–**4** were determined using a Synapt G2-S high-definition mass spectrometer (HDMS) with electrospray ionization (ESI) (Waters GmbH, Eschborn, Germany) coupled with an ACQUITY UPLC core system (Waters GmbH) according to the literature.^41^ For chromatography, a BEH C18 column (150 mm × 2.1 mm, 130 Å, 1.7 μm) with a corresponding guard column (Waters GmbH) and, as solvents, water (A) and acetonitrile (B), both with 0.1% formic acid, were used. The following gradient was used for the separation: starting with 5% B, increasing in 4.0 min to 100% B, and maintaining 100% B for 0.5 min. Other instrument parameters were obtained by Lang et al.^44^ For data acquisition and processing, MassLynx 4.1 SCN 8.5.1 (Waters GmbH) was used.

### Nuclear Magnetic Resonance Spectroscopy (NMR)

Structure elucidation was performed via one- (^1^H, ^13^C) and two-dimensional NMR measurements (H, H correlation spectroscopy (COSY)); H, C heteronuclear single-quantum coherence (HSQC); and H, C heteronuclear multiple-bond correlation (HMBC). For data acquisition, an AVANCE NEO 500 MHz Spectrometer equipped with a cryoprobe (CP 2.1 TCI 500 S2 H–C/N-D-05 Z XT) at 298 K (Bruker, Rheinstetten, Germany) was used. Data acquisition was performed using TopSpin 4.1.1 software, and data processing was done using TopSpin 4.0.9 (Bruker) and MestReNova 11.0.4 (Mestrelab Research, La Coruña, Spain).

#### Quantitative ^1^H NMR Spectroscopy (qHNMR)

An AVANCE 400 MHz III spectrometer equipped with a 5 mm BBI z-gradient probe (Bruker) was used to quantitatively determine the concentration of compounds **1**–**4** via qHNMR. The spectrometer was calibrated using external references: caffeine (3.58 mmol/L) and l-tyrosine (4.34 mmol/L) solutions. 1–2 mg of common compounds were put in 178 mm × 5 mm NMR tubes (Z172600 USC tubes, Bruker, Faellanden, Switzerland). The compounds were dissolved in 600 μL of D_2_O and quantified after manual phase, baseline adjustment, and signal integration. Software TopSpin 3.6 (Bruker) uses the ERETIC 2 (Electronic REference To access In vivo Concentrations) feature utilizing the PULCON (PULse length-based CONcentration) methodology for calculating the exact concentration of the compound solutions.^45^ All spectra were referenced to TMSP or the solvent signal.

### Sensory Analyses

#### Sensory Panel and Training

For all sensory analyses, 12–14 (female and male) trained panelists aged 22–35 from the Chair of Food Chemistry and Molecular Sensory Science of the Technical University of Munich without known taste disorders rated the given solutions. All sensory tests and training were performed under controlled conditions at room temperature in sensory booths with constant air conditioning and yellow light. Evian water with formic acid (pH 5.6–5.7) was used to prepare all sensory samples. For sensory panel training, all panelists received samples in a duo-trio test sample set for salty (NaCl; 20.0 mmol/L), bitter (caffeine; 1.0 mmol/L), sweet (sucrose; 50.0 mmol/L), and sour (lactic acid; 20.0 mmol/L) in water based on literature.^27,46^ The taste quality of umami and the taste impression of kokumi were trained in six 1:1 dilution steps (umami (MSG): 0.1–4.6 mmol/L; kokumi: (reduced glutathione in model broth) 0.3–10.8 mmol/L) by tasting from low to high concentration. For producing 500 mL model broth, NaCl (1.4 g), maltodextrin (3.2 g), yeast extract (1.0 g), and MSG (1.0 g) were dissolved in Evian water with the addition of formic acid (pH 5.6–5.7).^35,46,47^ All samples were coded randomly, and to avoid the ortho- and retro nasal perception, all panelists had to wear a nose clip during sensory evaluation.^27,48,49^

#### Intrinsic and Taste-Modulating Threshold

For the sensory evaluation of compounds **1**–**4**, the exact concentration and purification of a minimum of 98% were determined by qHNMR. To determine the intrinsic taste thresholds of **1**–**4**, a defined concentration was prepared in water (Evian, pH 5.6–5.7) and for the taste-modulating threshold in model broth (pH 5.6–5.7). The solutions were then diluted 1:1 with water or a model broth. The panelists evaluated the solutions in duo-trio tests (water or model broth as reference) from the lowest to the highest concentration. The geometric mean between the lowest concentration, where a difference is recognized as detectable between the blank and the spiked sample, and the last recognized concentration is described as the individual taste threshold. The whole panel’s taste threshold is calculated as the geometric mean of all individual taste thresholds.^50−52^

#### β-Value

For literature comparison, the so-called β-value of each compound was determined besides the taste thresholds. Therefore, a fixed solution (50 mmol/L) of each compound (**1**–**4**) was dissolved in an MSG solution (3 mmol/L). For comparison, solutions of logarithmic increasing intervals (30%) of IMP concentrations (50, 71, 102, 146, and 208 mmol/L) were produced in MSG solution (3 mmol/L). The fixed solutions of the nucleotide derivatives (**1**–**4**) were then evaluated against the increasing IMP solutions by paired choice comparison tests.^25,27,30^ The panelists had to decide which sample was the most intense in kokumi or umami. The consecutive, more intense fixed nucleotide derivate sample answers are evaluated and processed by statistical probit analysis via Microsoft Excel 2016 (Microsoft Corporation, Redmond) and R (Version 4.0.2, R Foundation).^53^ The resulting β-value based on the equation *v* = β·*v*′ (*v*: concentration of IMP; *v*′: concentration of test nucleotide derivate) of Yamaguchi et al.^30^ represents the ratio between the concentration of IMP and the test nucleotide at the equality point of umami intensity.^25,27,30^

## Results and Discussion

### Isolation and Structure Elucidation of *N*^2^-(Alkylthiomethyl)- and *N*^2^-(Arylthiomethyl)-Substituted GMP Derivatives

**Figure 1:**
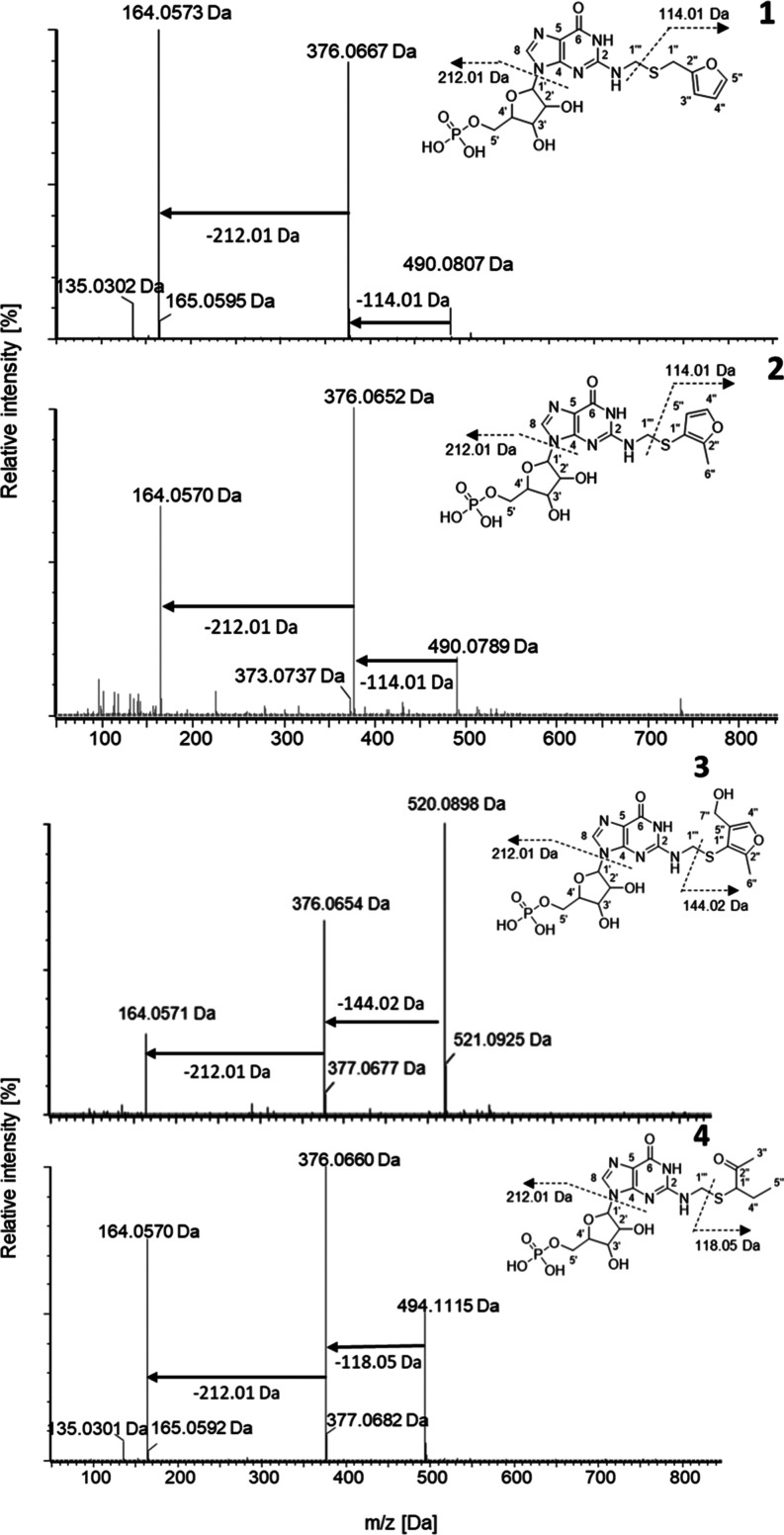
ESI^+^-UHPLC-ToF-MS^E^ spectra (HDMS) of the compounds **1**–**4** (**1**: model reaction of FFT, formaldehyde, GMP fraction five out of eight; **2**: MFT, formaldehyde, GMP fraction six out of eight; **3**: MFT, formaldehyde, GMP fraction five out of eight; **4**: MP, formaldehyde, GMP fraction six out of seven) using 20–60 eV ramp voltage (relative intensity [%]; mass-to-charge ratio (*m*/*z*) [Da]).

The formation of different *N*^2^-substituted GMP derivatives was implemented as a two-step model reaction in a sucrose/d-sorbitol/water (1:1:8) NADES system according to the generation of *N*^2^-(furfurylthiomethyl) guanosine 5′-monophosphate (**1**)^41^ with some modifications based on the literature.^27,43^ In the first reaction step, each aroma compound (FFT, MFT, or MP) was mixed and heated with the Maillard reaction product, formaldehyde, in the NADES system. After 4 h of reaction time at 40 °C, the second reaction step is induced by adding the GMP.^27,41^ After an additional 16 h at 40 °C, the mixture was entirely dissolved in water and fractionated via RP-HPLC-UV/vis into eight fractions for the MFT-, five for the MP-, and eight for the FFT model reaction mixture. All fractions obtained were screened via UHPLC-ToF-MS/MS in ESI^+^ and ESI^–^ ionization modes. The fragment ions of 164.06 and 376.07 Da in the ESI^+^ mode could be detected in different fractions of all model reaction mixtures (Figure 1), well in line with the assumption that a GMP and a formaldehyde moiety are somehow incorporated in the target molecules (**1**–**4**). The reaction products **1**–**4** could be identified by their pseudomolecular ions ([M + H]^+^; **1**, **2**: *m*/*z* 490.08; **3**: *m*/*z* 520.09; **4**: *m*/*z* 494.11) and the cleavage of respective thiol units of **1**, **2** (114.01 Da), **3** (144.02 Da), and **4** (118.05 Da), which all lead to the characteristic fragment ion of *m*/*z* 376.07, as described above.

Zappey et al. have already reported that the heterolytic cleavage of the carbon–sulfur bond belongs to the dominant reactions of thioethers in the mass spectrometer’s ion source.^54^ Furthermore, amines favor the so-called α-cleavage due to their strong electron-donating properties and ability to stabilize the nascent charge.^55^ The neutral fragmentation product of 212.01 Da probably belongs to the phosphate and sugar moiety. Strzelecka et al. found that for methylated GMP derivatives in the ESI^–^ mode, the fragment ion of 211.2 Da fits the observed phosphoribosyl neutral loss in the ESI^+^ mode.^56^ The remaining 2-*N*-methylated guanine moiety showed an intense fragment ion *m*/*z* of 164.06 Da. A further possible α-cleavage between C(2) and the amino group results in the observed fragment ion of 135.03 Da, which aligns with the purine moiety. This fragment ion of GMP derivatives was verified in the ESI^–^ mode with *m*/*z* 134.0 by Strzelecka et al.^56^ All isolated compounds (**1**–**4**) showed the typical fragmentation pattern of GMP derivatives, as described in the literature and, in addition, the expected fragments of the individual thiol moieties.

The MFT, formaldehyde, and GMP model reaction revealed the characteristic fragment ions of 376.07 and 164.06 Da in two different fractions. Fraction six showed the pseudomolecule ion of 490.08 Da and the elemental composition of C_16_H_21_N_5_O_9_PS^+^, as expected, whereas fraction five (**3**) showed a pseudomolecule ion of 520.09 Da in the ESI^+^ mode. The mass difference of 30 Da, as well as the variation of the elemental composition (C_17_H_23_N_5_O_10_PS^+^), most likely corresponds to an additional CH_2_O group. The additional group was separated in the MS^E^ spectrum by a cleavage of 144.02 Da, which indicates the connection of the CH_2_O group to the MFT moiety. To verify all of these assumptions, one- and two-dimensional NMR experiments were performed to elucidate the constitution of target compounds **1**–**4**.

All ^1^H and ^13^C signals observed by NMR spectroscopy and the mass spectrometric data fit with the literature data of **1**, and therefore, the compound could be identified as the previously reported *N*^2^-(furfurylthiomethyl)-guanosine 5′-monophosphate.^27,41^

The ^1^H NMR spectrum of compound **2** showed a total of 11 signals: one methyl group at 2.28 ppm as a singlet, two multiplets for the protons between 4.11 and 4.25 ppm, two triplets for the protons at 4.38 and 4.62 ppm, five doublets of each integrated for one proton at 4.59, 4.72, 5.93, 6.43, and 7.34 ppm, and one singlet at 8.25 ppm. One characteristic signal of the MFT moiety is the methyl group at H C(6″) (2.28, 11.7 ppm) attached to the furan ring at position H–C(2″) (157.2 ppm). The furan ring showed two direct adjacent aromatic protons at 6.43 and 7.34 ppm with a typical coupling constant of 1.9 Hz verified by H, H-correlations in the COSY spectrum. Due to the electronegativity of the oxygen atom, H–C(4″) (7.34, 142.3 ppm) is deshielded and therefore shifted to higher frequencies compared to the H–C(5″) (6.43, 116.5 ppm).^32^

**Figure 2:**
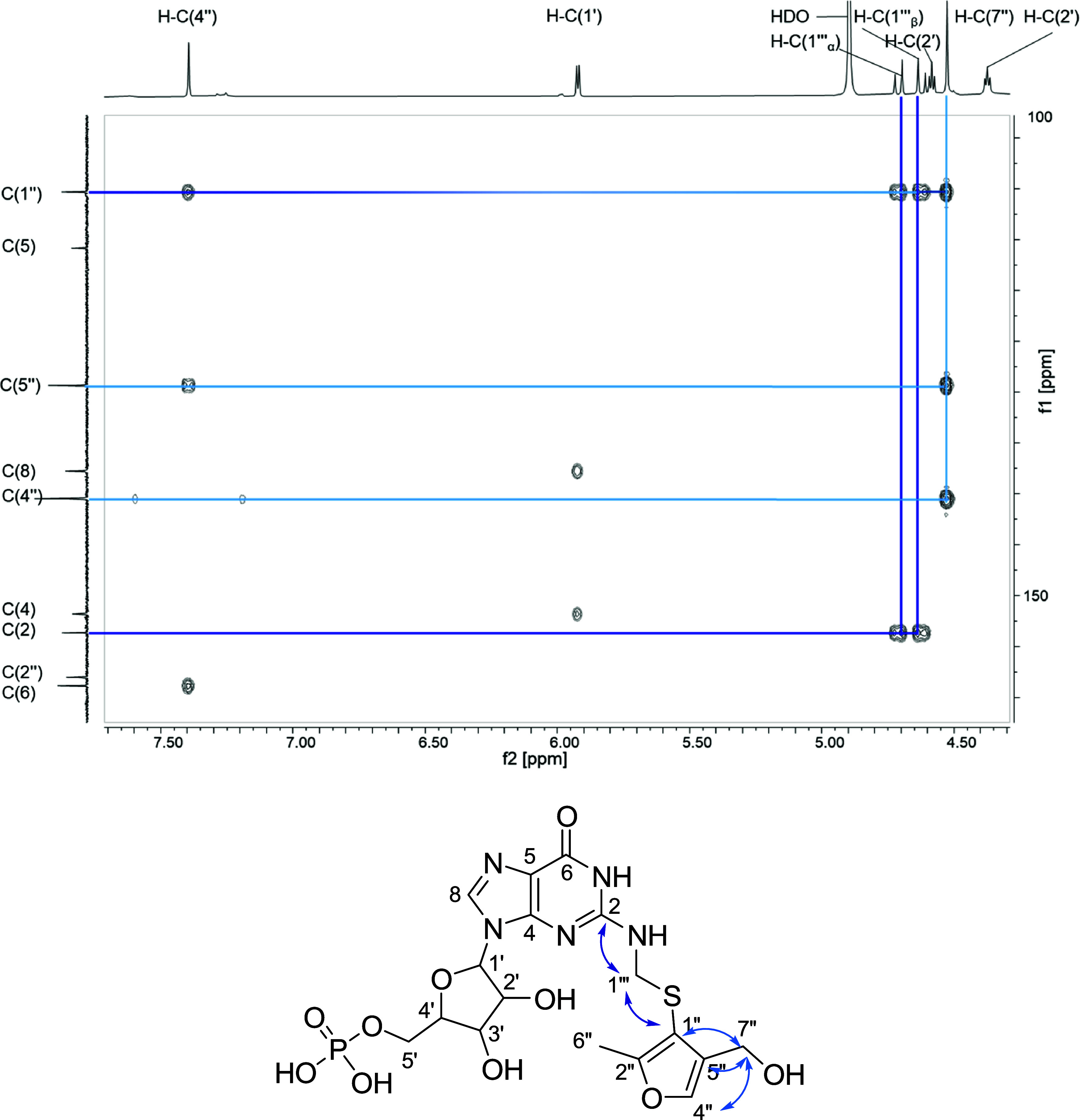
Excerpt of the H,C HMBC spectrum (500 MHz, 125 MHz, methanol-*d*_4_, 298 K) of compound **3** with significant correlations.

The protons of the sugar moiety were verified by ^3^*J*-correlations in the H, H–COSY spectrum. The anomeric proton H–C(1′) (5.93 ppm, d, *J* = 5,2 Hz) showed a correlation to H–C(2′) (4.62 ppm) as well as H–C(2) to H–C(3′) (4.38 ppm). The multiplet between 4.21 and 4.25 ppm (H–C(4′)) showed connectivity to a diastereotopic methylene group between 4.11 and 4.21 ppm, which could be assigned as (H–C(5′)). The corresponding carbon atoms 64.7 ppm (C(5′)), 71.9 ppm (C(3′)), 75.9 ppm (C(2′)), 85.2 ppm (C(4′)), and 89.5 ppm (C(1′)) were assigned by ^1^*J*_C,H_ couplings in the HSQC spectrum. The connection between the sugar moiety and the purine ring, indicating the intact GMP, was verified by ^3^*J*_C,H_-couplings between H–C(1′) and C(8) (138.1 ppm) or rather C(4) (152.1 ppm) in the HMBC spectrum. Moreover, according to the literature, the phosphate group attached to the pentose could be detected by the coupling of the ^31^P with the carbon atoms at position C(4′) and C(5′) through the ^2^*J*_C,P_ and ^3^*J*_C,P_ coupling constants of 5.3 and 8.5 Hz.^27^ In addition, in the HMBC spectrum, H–C(8) at 8.25 ppm showed ^3,4^*J*_C,H_ correlations with the quaternary C atoms at 116.7 (C(5)) and 158.5 ppm (C(6)), well in line with the assumption of the intact purine moiety. Due to a lack of correlations within the purine ring, the carbon atom C(2) was assigned via the coupling to the diastereotopic methylene groups H–C(1‴_α_) (4.59 ppm) and H–C(1‴_β_) (4.72 ppm) (Figure 2). The key ^3^*J*_C,H_ correlations of the diastereotopic methylene group H–C(1‴_α_) and H–C(1‴_β_) with C(1″) (110.0 ppm) as well as C(2) clearly demonstrates the connection of GMP via H–C(1‴_α/β_) to the MFT moiety. This structural feature was observed in all isolated GMP derivatives (**1**–**4**). Considering all of these spectroscopic and spectrometric data, compound **2** could be identified as *N*^2^*-(*2-Methyl-1-furylthiomethyl)-guanosine 5′-monophosphate (**2**). To the best of our knowledge, this compound has not yet been described in the literature.

Three differences could be observed when comparing the ^1^H NMR spectra of fractions five and six of the model reaction GMP, formaldehyde, and MFT (Figure S7, Supporting Information). In contrast to **2**, an additional singlet with an integral of two protons resonating at 4.53 ppm appeared in the spectrum of fraction five, whereas the signal of H–C(5″) was no longer detectable. Consequently, the doublet of H–C(4″) changed into a singlet. The assumption that C(5″) was transformed into a tertiary C-Atom by side reactions was verified by the observed ^2,3^*J*_C,H_ correlations of the additional methylene group (H–C(7″)) with C(5″), C(4″) and C(1″) in the HMBC spectrum (Figure 2). Compound **3** is most likely formed by the nucleophilic addition of a second formaldehyde molecule at position C(5″) of the MFT moiety. Taking all MS- and NMR data into account, compound **3** could be unequivocally identified as *N*^2^*-*((5-Hydroxymethyl)-2-methyl-1-furylthiomethyl)-guanosine 5′-monophosphate (**3**), which was not described in the literature until now.

MP, an essential key aroma compound in meat and yeast extract,^57^ was used the same way as MFT and FFT to produce potentially new taste-modulating GMP derivatives in NADES systems. The target compound showed a [M + H]^+^ of *m*/*z* 494.11 and a corresponding elemental composition of C_16_H_25_N_5_O_9_PS^+^. Due to the chiral carbon C(1″) in MP and the fact that the starting material was not enantiomerically pure, the formation of diastereomers will occur during the model reaction, which could be obtained by a double signal set in the NMR spectra of the target compound (Figure S8, Supporting Information). Unfortunately, it was impossible to separate the two compounds in sufficient purity by chromatography for signal assignment of the individuals; therefore, structure elucidation was performed with the mixture of the diastereomers (ratio approximately 1:1).

According to the other GMP derivatives (**1**–**3**), in the ^1^H NMR spectrum, the signals of the sugar moiety, the purine ring, and the “linker” methylene group showed similar chemical shifts compared to the compounds **1**–**3**. Furthermore, the proton NMR shows additional signals at 0.98, 1.66, 1.76, 1.84, 1.96, 2.28, and 3.55 ppm. In the H,H–COSY spectrum, the triplet at 0.98 ppm (*J* = 7.4 Hz), which was assigned as H–C(5″), showed ^3^*J*_H,H_ correlations to H–C(4″) and the latter to H–C(1″). H–C(4″) could be assigned as a diastereotopic methylene group by ^1^*J*_C,H_ couplings in the phase-sensitive H,C-HSQC spectrum, showing two separated negative phase correlation signals with the carbon signal at 23.5 ppm. In the HMBC experiment, ^2^*J*_C,H_ correlations of H–C(1″) as well as methyl group H–C(3″) to keto group C(2″) at 207.1 ppm could be observed. In addition, ^3^*J*_C,H_ correlations of H–C(4″) to C(2″) and H–C(5″) to C(4″) confirmed the MP motif in the suggested structure. The connectivity of the purine ring and the thiol compound via the “linker” methylene group, derived initially from formaldehyde, was identical to **1**–**3**. Based on the MS- and NMR data, compound **4** could be identified as *N*^2^-((2-pentanon-1-yl)thiomethyl)-guanosine 5′-monophosphate (**4**).

In summary, four compounds (**1**–**4**) were successfully formed via model reactions of GMP, formaldehyde, and different aroma-active thiols (FFT, MFT, and MP) in NADES and completely characterized via LC-ToF-MS/MS and one- and two-dimensional NMR experiments. To the best of our knowledge, three of these four compounds (**2**–4) have not been reported so far in the literature.

### Formation Pathway and Structure Elucidation of Intermediates

**Figure 3:**
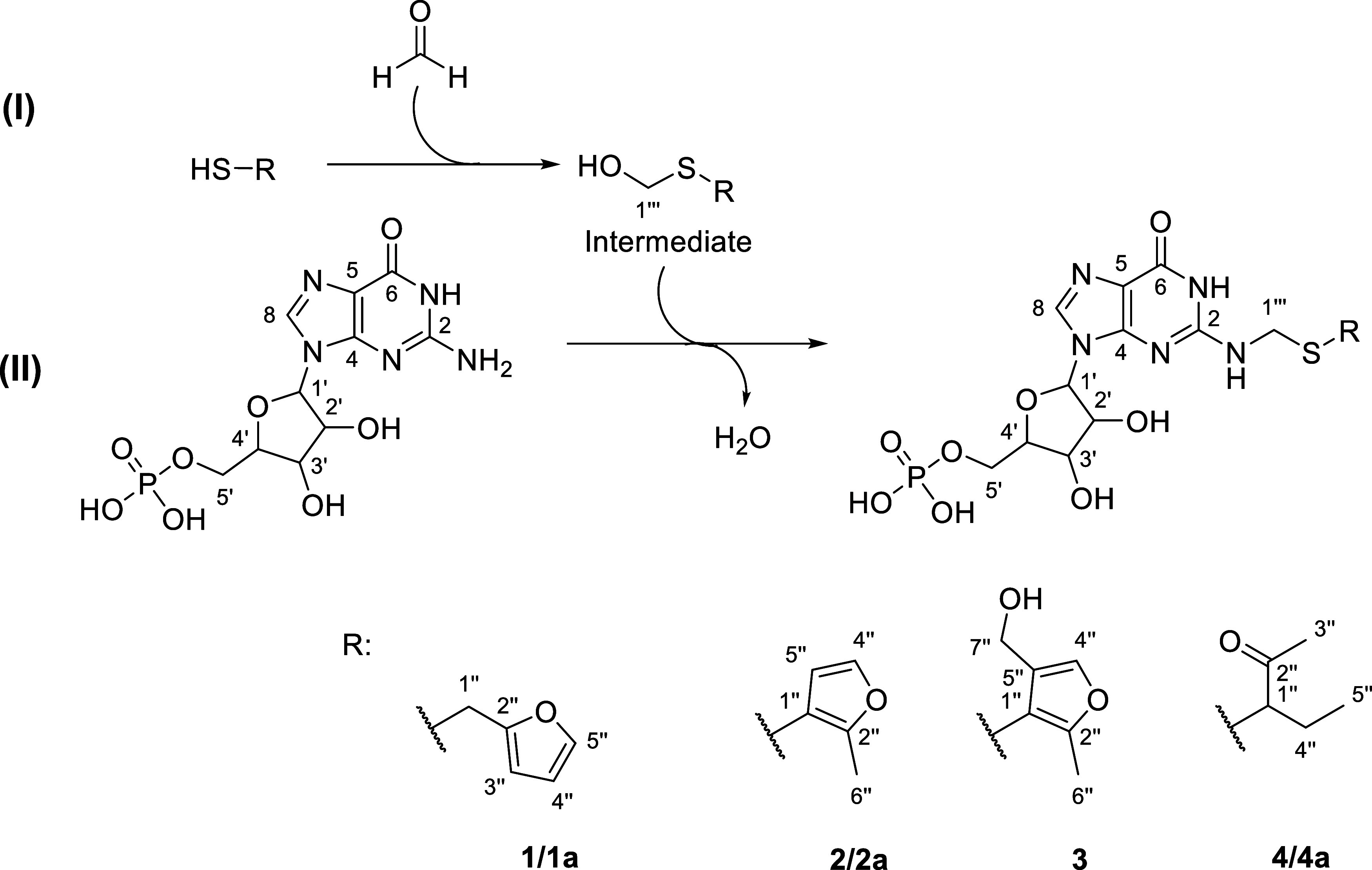
Formation pathway of the Maillard-type reaction of formaldehyde, the aroma-active thiols (FFT, MFT, MP), and GMP for forming *N*^2^-Alkyl- and *N*^2^-arylthiomethylated GMP derivatives (adapted from Suess et al.^27^).

In accordance with Suess et al., the two-step reaction pathway for the formation of these Maillard-type reaction products is displayed in Figure 3.^27^ The first reaction step characterizes the nucleophilic addition of the individual thiol derivative to formaldehyde by the formation of the respective methylthio-intermediate (I). The second reaction step is a nucleophilic substitution of the intermediate with GMP under loss of water.^27^ Formaldehyde operates as an electrophilic linker between GMP and aroma-active thiol (II).

After the first reaction step of each model reaction, the respective intermediates generated by formaldehyde and the individual thiol compounds were isolated by extraction with ethyl acetate. After removing the solvent, the residues were dissolved in DMSO-*d*_6_, and the structures were analyzed via one- and two-dimensional NMR experiments. The signal assignment of the thiol moieties was comparable to that of the corresponding structural element in the respective GMP derivatives (**1**–**4**). The additional hydroxymethyl group connected as a thioether was verified in each intermediate by the heteronuclear ^2,3^*J*_C,H_ coupling of the methylene group at position 1‴ to C(1″) in the HMBC-spectra. The intermediates 2-furfurylthiomethanol (**1a**), 2-methyl-3-furanthiomethanol (**2a**), and 3-hydroxymethylthio-2-pentanone (**4a**) of compounds **1**, **2**, and **4** could be confirmed, and all signals could be assigned (Supporting Information). In addition, the connection between each thiol C(1″) (**1a**, **2a**, **4a**) and H–C(1‴) originating from formaldehyde demonstrated the formation of the so-called hemithioacetals. Generally, hemithioacetals or hemithioketals can be formed under mild conditions through acid or specific base catalyzation.^58^ Their stability was explained by resonance stabilizing effects. The reactive species that creates hemithioacetals is the unhydrated carbonyl compound.^58^ Interestingly, the intermediate **3** could not be generated. This observation supports the assumption that adding the second molecule of formaldehyde to the MFT is subject to different reaction kinetics compared to the formation of **2a**. The intermediate **3** may be formed during the second reaction step or after GMP addition, which may impact the electron density of the furan ring. Nevertheless, for **1**, **2**, and **4**, the formation pathway, including the hemithioacetal formation suggested by Suess et al.,^27^ was verified by isolation of the intermediates **1a**, **2a**, and **4a**.

### Psychophysical Studies

Since a positive modulating synergistic effect between various GMP derivatives and MSG has been described in the literature,^16,26,27^ the new compounds will also be studied for their taste-modulating properties. To determine taste modulating or taste active properties of **1**–**4**, all compounds were sensorially evaluated after ensuring a minimum purity of 98% via qHNMR measurements.^50,59^ Using a duo-trio sensory test setup, the sensory panel evaluated each derivative in water (pH 5.6) for intrinsic taste and model broth (pH 5.6) for taste-modulating effects in ascending concentrations. In addition to the taste thresholds, so-called β-values were evaluated using a 50 mmol/L solution of the individual GMP derivative (**1**–**4**) compared to different solutions of increasing IMP concentrations. Generally, the β-values are numerical factors for representing the relative flavoring activity, especially the taste-modulating effect of any nucleotide compared to IMP. This means that the higher the β-value, the stronger the synergistic, taste-modulating impact of the test nucleotide.^30^ All determined sensory values of compounds **1**–**4**, IMP, and GMP are listed in Table 1. In principle, the nucleotide IMP shows no intrinsic umami taste. Yamaguchi^60^ proved that the l-glutamate concentration in human salvia causes the slight intrinsic taste of nucleotides like IMP.^60^ Taking these results into account, potential intrinsic taste thresholds were determined based on practical considerations. Intrinsic umami concentrations of 884 and 468 μmol/L were determined by the sensory panel for IMP and GMP, respectively. In the literature, an umami threshold in water of 1000–4000 μmol/L is described for IMP.^24,61^ Yamaguchi^60^ published an umami threshold for IMP of 630 μmol/L. Our in-house-determined threshold of 884 μmol/L is in the range of these different values. Festring and Hofmann^62^ established an umami recognition threshold of 150 μmol/L for the disodium salt of GMP, which is below the determined intrinsic threshold of 468 μmol/L. The difference can be explained by using the disodium salt of GMP by Festring and Hofmann.^62^ For the GMP derivatives (**1**–**4**), intrinsic umami thresholds between 107 μmol/L (**2**) and 128 μmol/L (**1**) and intrinsic astringent thresholds between 141 μmol/L (**2**) and 178 μmol/L (**4**) could be determined.

**Table 1:** Taste Threshold Concentrations in μmol/L of Ribonucleotides IMP, GMP, and the Compounds **1**–**4** in Water, Model Broth, and β-Values According to Yamaguchi et al.^30^

	taste threshold concentration [μmol/L]		
compound	in water	in model broth	β-value^30^
IMP	884 (umami)	41 (umami)	1.0^27,30^
GMP	468 (umami)	26 (umami)	2.1–2.4^26−28,30^
**1**	128 (umami)	19 (umami/kokumi)	2.8
	166 (astringent)		
**2**	107 (umami)	20 (umami/kokumi)	2.3
	141 (astringent)		
**3**	119 (umami)	22 (umami/kokumi)	2.7
**4**	178 (astringent)	21 (umami/kokumi)	2.2

In addition, all isolated GMP derivatives **1**–**4** showed taste-modulating effects between 19 μmol/L (**1**) and 22 μmol/L (**3**) regarding umami or rather kokumi sensations in model broth, well in line with literature-known synergistic effect of nucleotides and MSG.^18^ The newly identified compounds showed values below the umami taste-modulating thresholds of the pure nucleotides IMP (41 μmol/L) and GMP (26 μmol/L) in model broth and therefore promise a higher potency in terms of taste enhancement. In good agreement with the taste-modulating thresholds, the β-values also show similar trends by the determined values ranging from 2.2 (**4**) to 2.8 (**1**), well in line with the β-value published by Suess et al. of 3.1 for compound **1**.^27^ Compound **1**, with the highest β-value of 2.8, also showed the lowest modulating threshold of 19 μmol/L and showed higher taste-modulating activity than IMP.

Since the β-value is a multiplicative factor compared to the taste-modulating effect of IMP, the modulating taste threshold value of, e.g., compound **1** can be theoretically calculated using the determined modulating taste threshold value of IMP (41 μmol/L) and the determined β-value of **1** (2.8). So, recalculating for the modulating threshold means 41 μmol/L divided by 2.8, resulting in 14.6 μmol/L for **1**. Compared to the determined modulating taste threshold of 19 μmol/L for **1**, there is only a minor difference of 4.4 μmol/L. Therefore, the determined values for the taste-modulating activity of the four GMP derivatives **1**–**4** could be confirmed by two independent sensory tests (β-value, modulating threshold determination). The furan ring of **1**–**3** reduces the threshold or increases the β-value slightly compared to **4.** In contrast to the literature, Suess et al.^27^ showed that adding an aromatic phenyl group reduced the β-value from 5.1 to 2.7. Cairoli et al.^26^ observed the same effect with a reduction from 4.1 to 2.9. Obviously, the size and constitution of the aromatic residue seemed to play an essential role in the synergistic taste-modulation effect, well reflected by the fact that **1** showed a lower threshold value than its constitutional isomer **2**.

In summary, four different GMP derivatives (**1**–**4**) could be isolated from the model reaction mixtures of GMP, formaldehyde, and the naturally occurring aroma-active thiols FFT, MFT, and MP. Compounds **2**–**4** were isolated and completely characterized for the first time via LC-ToF-MS/MS and one- and two-dimensional NMR measurements. In addition, the two-step formation pathway could be confirmed by isolation and structure verification of the hemithioacetals **1a**, **2a**, and **4a** as precursors of **1**, **2**, and **4**. Furthermore, all intrinsic umami thresholds of compounds **1**–**4** ranging from 107 to 128 μmol/L are lower than those of IMP (883 μmol/L) and GMP (468 μmol/L), and all modulating thresholds ranging from 19 to 22 μmol/L are below the intrinsic thresholds. In addition, the synergistic effect of compounds **1**–**4** was verified by the determination of so-called β-values (2.2–2.8). Consequently, the investigated GMP derivatives **1**–**4** show high potential as taste-modulating compounds, e.g., for intensifying vegetarian and vegan food flavors. Further investigations will show that GMP derivatives, such as Maillard reaction products, could potentially reduce processed foods’ salt and MSG content by production in food-grade model reactions. Therefore, upscaling to an industrial scale and using these novel taste-modulating substances in complex food matrices must be investigated in the next step. These results may contribute to a better understanding of structure–activity relationships of nucleotide synergism and umami taste, as well as understanding further reactions of aroma and taste compounds in the Maillard reaction in the future.

## Supporting Information Available

Molecular structure of literature-known umami taste-modulating nucleotides; NMR signal assignment of the intermediates **1a**, **2a**, and **4a**; chromatograms for isolation and purification of compounds **1**–**4**; comparison of ^1^H NMR spectra of compounds **2** and **3**; ^1^H NMR spectra of compound **4** (PDF)
