# Supplementary material for: Isolation and Identification of Novel Taste-Modulating N2-Guanosine 5′-Monophosphate Derivatives Generated by Maillard-Type Reactions
Source: J Agric Food Chem. 2024 Jun 13;72(25):14284–93. doi: 10.1021/acs.jafc.4c03485 (PMC11212044; doi:10.1021/acs.jafc.4c03485)
Supplement: Supplementary file 1 — jf4c03485_si_001.pdf [file jf4c03485_si_001.pdf]

Supporting Information

Isolation and identification of novel taste-modulating *N*²-guanosine 5'-monophosphate derivatives generated by *Maillard*-type reactions

Daniela M. Hartl[1], Oliver Frank[1*], Victoria S. Hänel[1], Vinzenz Heigl[1], Corinna Dawid[1,2], and Thomas F. Hofmann[1]

[1]Chair of Food Chemistry and Molecular Sensory Science, Technical University of Munich, Lise-Meitner-Str. 34, 85354 Freising, Germany

[2]Professorship for Functional Phytometabolomics, TUM School of Life Sciences, 10 Technical University of Munich, Lise-Meitner-Str. 34, D-85354 Freising, Germany

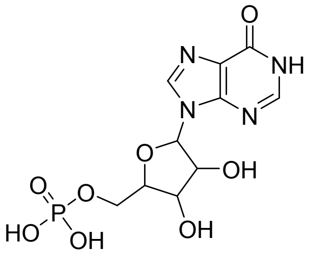

inosine 5'-monophosphate (IMP) (*1.0*)

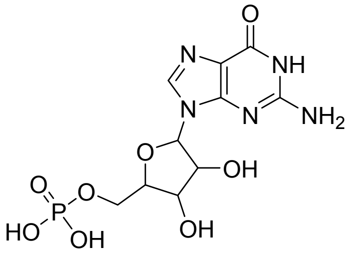

guanosine 5'-monophosphate (GMP) (*2.1-2.4*)

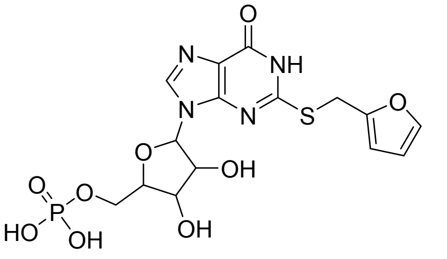

2-furfurylthioinosine 5'-monophosphate (*17.3*)

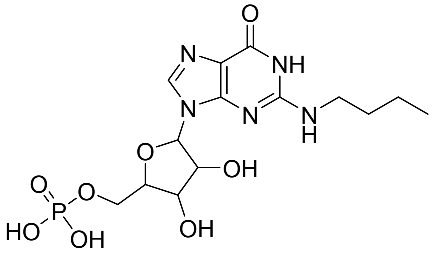

N^2-Butylguanosine 5'-monophosphate (*4.1*)

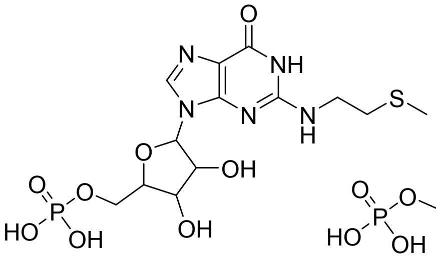

N^2-(methylthioethyl)guanosine 5'-monophosphate (*4.6*)

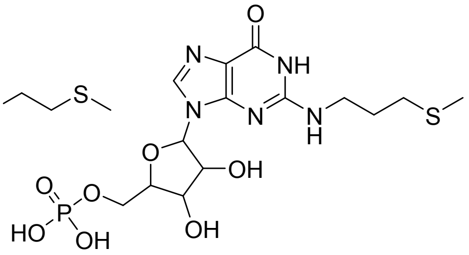

N^2-(methylthiopropyl)guanosine 5'-monophosphate (*5.7*)

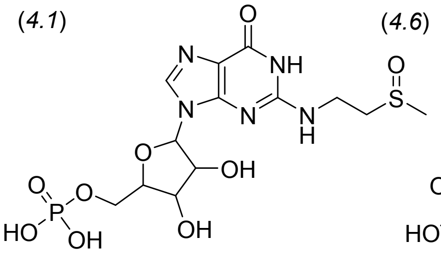

N^2-(2-Methylthioethyl)-5'-guanylic acid S-oxide (*2.9*)

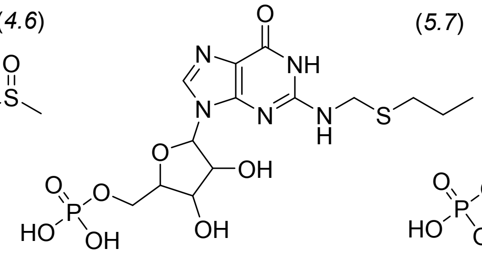

N^2-(propylthiomethyl)guanosine 5'-monophosphate (*5.1*)

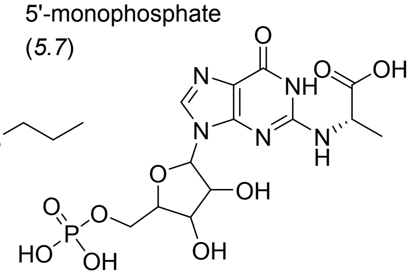

(*S*)-*N*^{2}-(1-carboxyethyl)-guanosine 5'-monophosphate (*7.0*)

Figure S1. Molecular structure of umami taste modulating nucleotides inosine 5'-monophosphate (IMP), guanosine 5'-monophosphate (GMP) and their derivatives 2-furfurylthioinosine 5'-monophosphate, [1] *N*^{2}-Butylguanosine 5'-monophosphate, *N*^{2}-(methylthioethyl)guanosine 5'-monophosphate, *N*^{2}-(methylthiopropyl) guanosine 5'-monophosphate, [2] *N*^{2}-(2-Methylthioethyl)-5'-guanylic acid s-oxide, *N*^{2}-(propylthiomethyl) guanosine 5'-monophosphate, [3] and (*S*)-*N*^{2}-(1-carboxyethyl)-guanosine 5'-monophosphate [4] with their corresponding *β*-values.

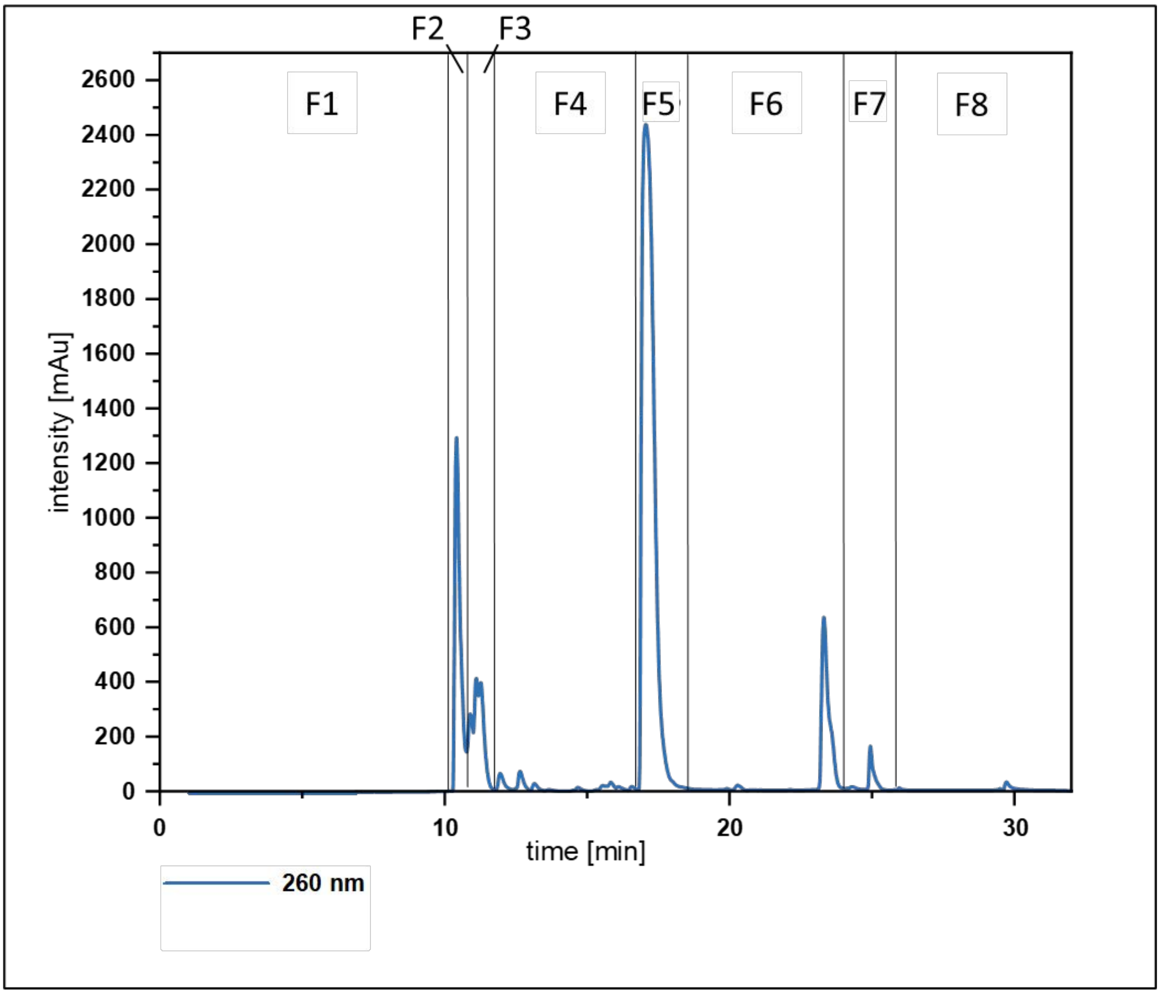

Figure S2: RP-HPLC-UV/Vis-chromatogram of the preparative separation at 260 nm of the *Maillard*-type model reactions of 5'-GMP, formaldehyde, and FFT (F1: D-sorbitol, sucrose; F2, F3: 5'-GMP, guanosine; F5: *N*[2]-(furfurylthiomethyl)-guanosine 5'-monophosphate (**1**)).

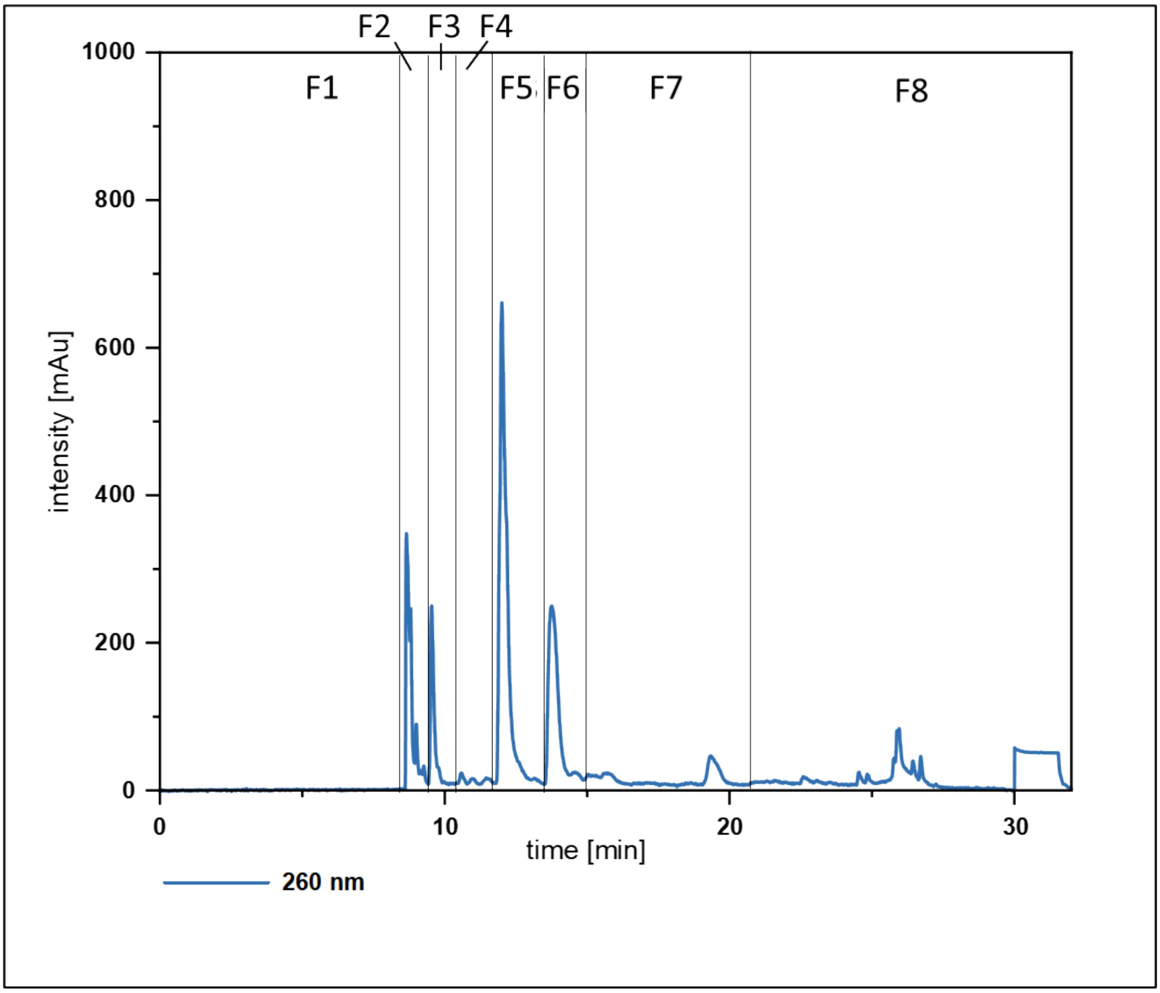

Figure S3: RP-HPLC-UV/Vis-chromatogram of the preparative separation at 260 nm of the *Maillard*-type model reactions of 5'-GMP, formaldehyde, and MFT (F1: D-sorbitol, sucrose; F2, F3: 5'-GMP, guanosine; F5: *N*^{2}-((5-Hydroxymethyl)-2-methyl-1-furylthiomethyl)-guanosine 5′-monophosphate (**3**); F6: *N*^{2}-(2-Methyl-1-furylthiomethyl)-guanosine 5′-monophosphate (**2**).

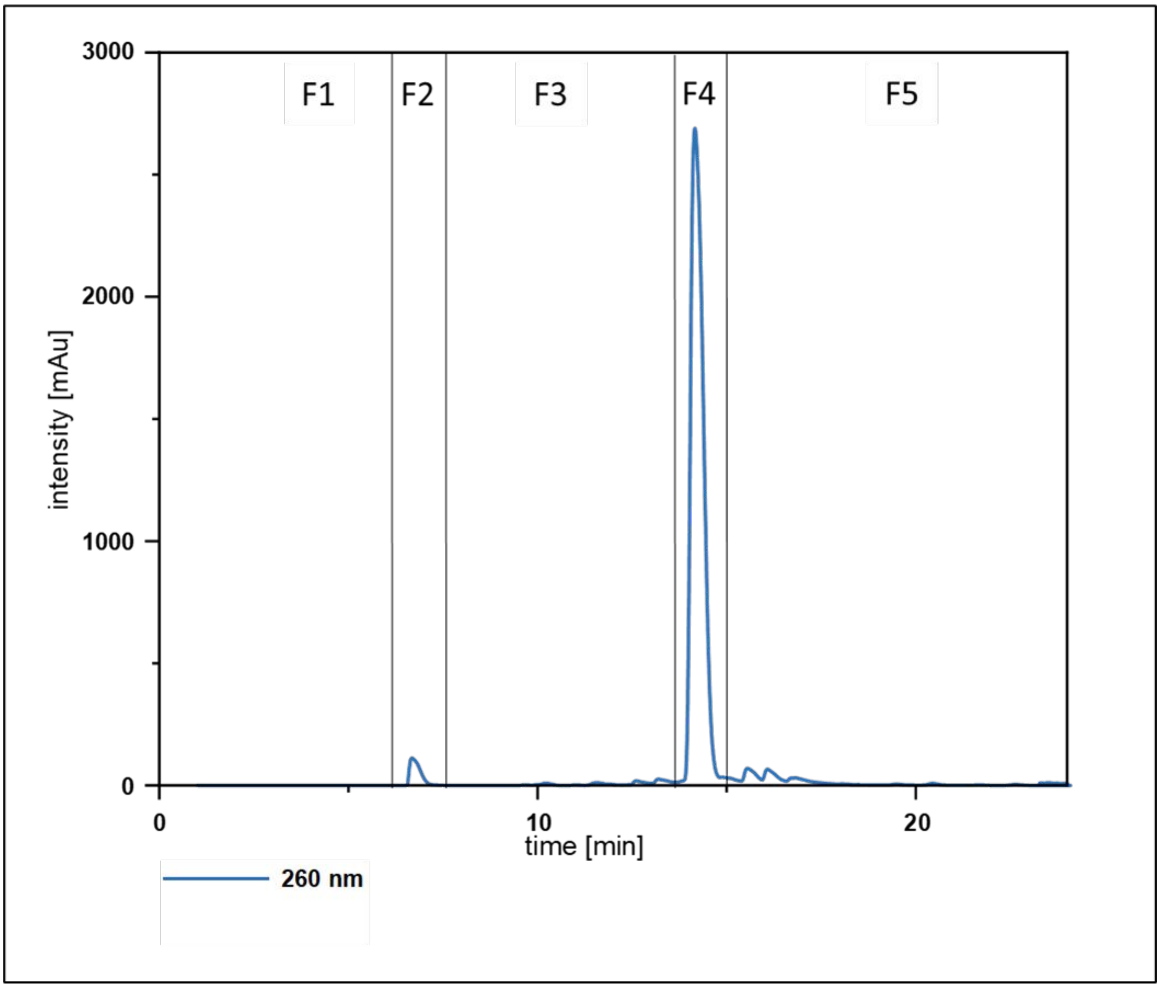

Figure S4: RP-HPLC-UV/Vis-chromatogram of the semi-preparative purification of fraction 5 of the *Maillard*-type model reactions of 5'-GMP, formaldehyde, and MFT at 260 nm (F4: *N*^{2}-((5-Hydroxymethyl)-2-methyl-1-furylthiomethyl)-guanosine 5′-monophosphate (**3**)).

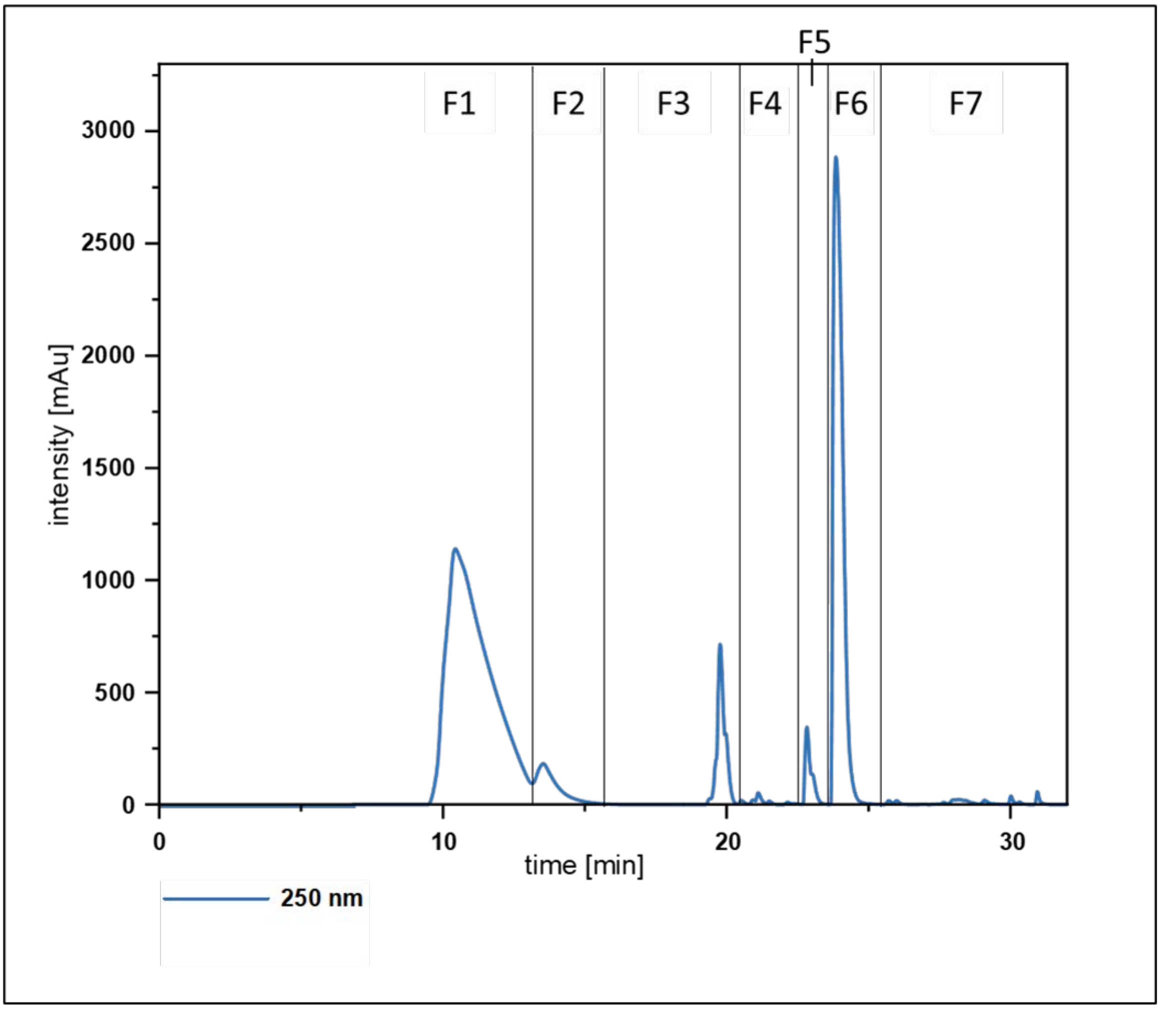

Figure S5: RP-HPLC-UV/Vis-chromatogram of the preparative separation at 250 nm of the *Maillard*-type model reactions of 5'-GMP, formaldehyde, and MFT (F1: D-sorbitol, sucrose; 5'-GMP; F2: guanosine; F6: (*R*)-, (*S*)-*N*^{2}-((2-Pentanon-1-yl)thiomethyl)-guanosine 5′-monophosphate (**4**).

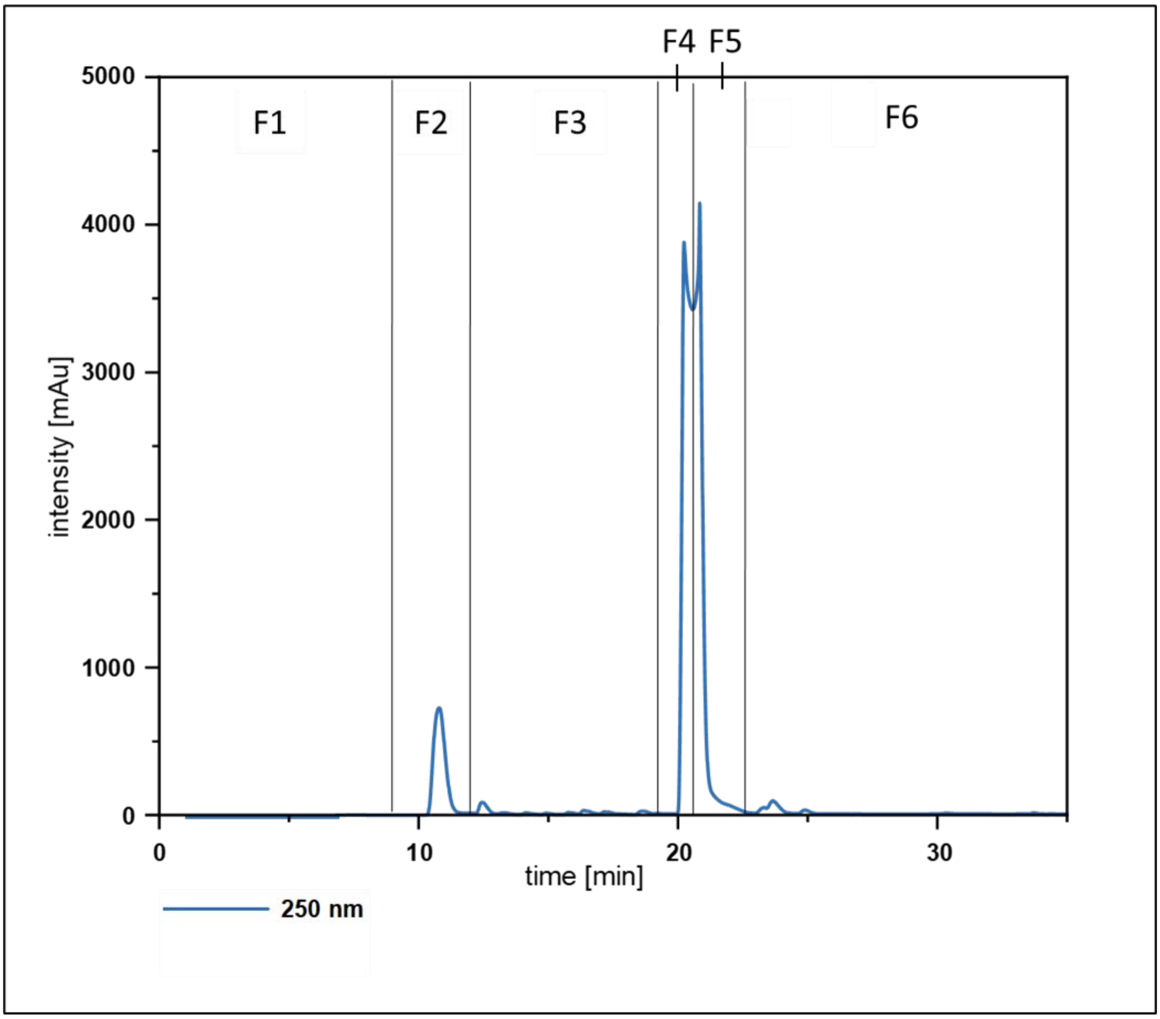

Figure S6: RP-HPLC-UV/Vis-chromatogram of the semi-preparative purification of F6 of the *Maillard*-type model reactions of 5'-GMP, formaldehyde, and MP at 250 nm (F4/F5: (*R*)-, (*S*)-N^2-((2-Pentanon-1-yl)thiomethyl)-guanosine 5′-monophosphate (**4A/4B**).

Intermediates:

2-Furfurylthiomethanol (**1a**). ^{1}H-NMR (500.13 MHz, DMSO-d_6, 298 K, COSY) δ (ppm) 3.84 [s, 2 H, H-C(1″)], 4.58 [d, 2 H, J = 7.2 Hz, H-C(1‴)], 5.86 [t, 1 H, J = 7.2 Hz, H-O], 6.26 [d, 1 H, J = 3.2 Hz, H-C(3″)], 6,36 [dd, 1 H, J = 1.9, 3.2 Hz, H-C(4″)], 7.55 [dd, 1 H, J = 0.9, 1.9 Hz, H-C(5″)]. ^{13}C-NMR (125 MHz, DMSO-d_6, 298 K, HMBC, HSQC) δ (ppm) 25.3 [CH_2, C(1″)], 64.1 [CH_2, C(1‴)], 107.6 [CH, C(3″)], 110.7 [CH, C(4″)], 142.6 [CH, C(5″)], 151.9 [C, C(2″)].

2-Methyl-3-furanthiomethanol (**2a**). ^{1}H-NMR (500.13 MHz, DMSO-*d*$_6$, 298 K, COSY) δ (ppm) 2.28 [s, 3 H, H-C(6″)], 4.67 [d, 2 H, *J* = 7.3 Hz, H-C(1‴)], 6.00 [t, 1 H, J = 7.5 Hz, H-O], 6.48 [d, 1 H, *J* = 1.8 Hz H-C(5″)], 7.54 [d, 1 H, *J* = 1.8 Hz H-C(4″)]. ^{13}C-NMR (125 MHz, DMSO-*d*$_6$, HMBC, HSQC) δ (ppm) 11.7 [CH_3, C(6″)], 68.6 [CH_2, C(1‴)], 110.0 [C, C(1″)], 115.0 [CH, C(5″)], 141.1 [CH, C(4″)], 153.6 [C, C(2″)].

3-Hydroxymethylthio-2-pentanone (**4a**). ^{1}H-NMR (500.13 MHz, DMSO-*d*$_6$, 298 K, COSY) δ (ppm) 0.82 – 0.95 [m, 3 H, H-C(5″)] 1.44 – 1.64 [m, 1 H, H-C(4a″)] 1.68 – 1.85 [m, 1 H, H-C(4b″)], 2.18 – 2.21 [m, 3 H, H-C(3″)], 3.43 [t, 1 H, *J* = 7.5, H-C(1″)], 4.60 [dd, 2 H, *J* = 0.9, 7.3 Hz, H-C(1‴)], 5.91 [t, 1 H, *J* = 7.2, H-O]. ^{13}C-NMR (125 MHz, DMSO-*d*$_6$, 298 K, HMBC, HSQC) δ (ppm) 11.8 [CH_3, C(5″)], 23.8 [CH_2, C(4″)], 26.8 [CH_3, C(3″)], 53.9 [CH, C(1″)], 64.5 [CH_2, C(1‴)], 205.8 [C, C(2″)].

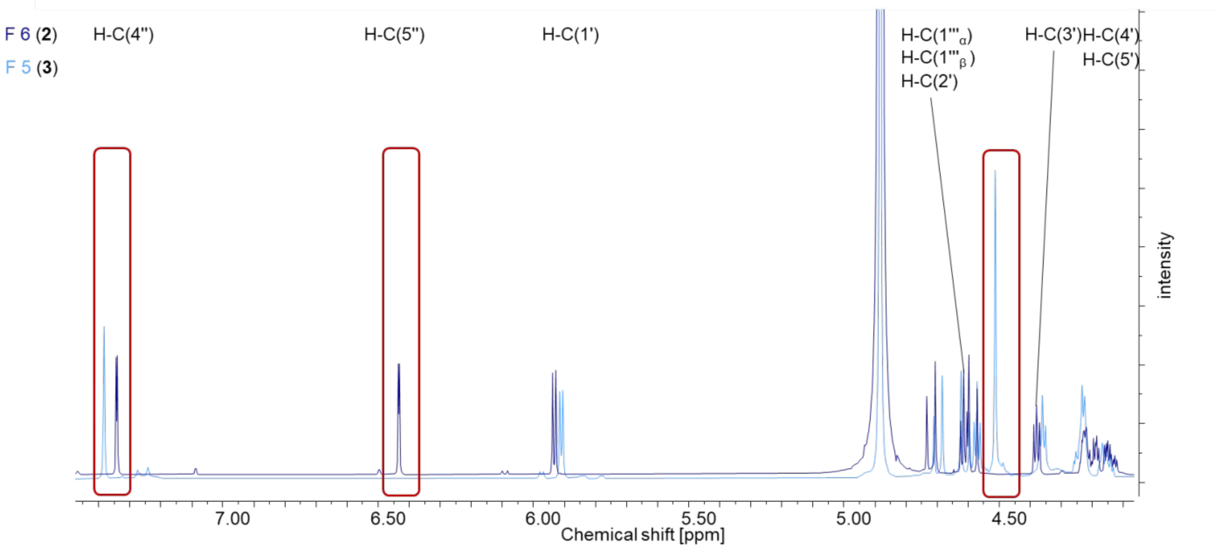

Figure S7. Excerpts of ^{1}H-NMR-spectra of compound **2** (fraction 6; dark blue; zg30, methanol-d_4, 500 MHz, 298 K) and compound **3** (fraction 5; light blue; zg30, D_2O, 500 MHz, 298 K); differences marked in red.

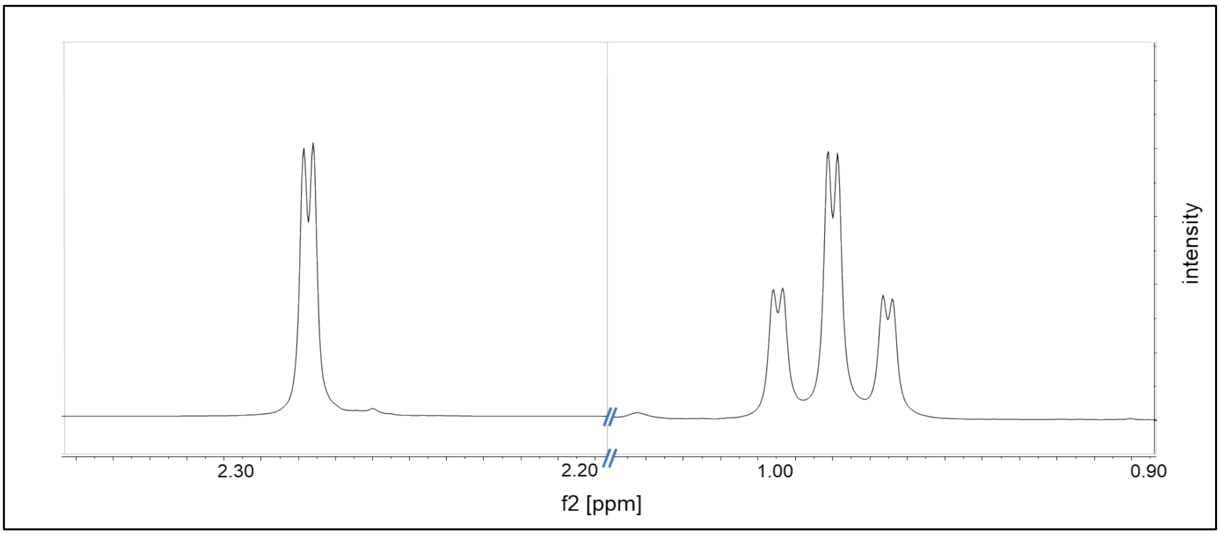

Figure S8: Excerpts of ^{1}H-NMR-spectra of compound 4 (zg30, methanol-d_4, 500 MHz, 298 K), signal H-C(5'') at 0.98 ppm and signal H-C(1'') at 2.28 ppm with their finestructure.

1. Imai, K.; Marumoto, R.; Kobayashi, K.; Yoshioka, Y.; Toda, J.; Honjo, M., Synthesis of compounds related to inosine 5'-phosphate and their flavor enhancing activity. IV. 2-Substituted inosine 5'-phosphates. *Chem. Pharm. Bull.* **1971,** *19* (3), 576-86.
2. Cairoli, P.; Pieraccini, S.; Sironi, M.; Morelli, C. F.; Speranza, G.; Manitto, P., Studies on umami taste. Synthesis of new guanosine 5′-phosphate derivatives and their synergistic effect with monosodium glutamate. *J. Agric. Food Chem.* **2008,** *56* (3), 1043-1050.
3. Morelli, C. F.; Manitto, P.; Speranza, G., Study on umami taste: the MSG taste-enhancing activity of N2-alkyl and N2-alkanoyl-5′-guanylic acids having a sulfoxide group inside the N2-substituent. *Flavour Fragr. J.* **2011,** *26* (4), 279-281.
4. Festring, D.; Hofmann, T., Systematic Studies on the Chemical Structure and Umami Enhancing Activity of Maillard-Modified Guanosine 5'-Monophosphates. *J. Agric. Food Chem.* **2011,** *59* (2), 665-676.
